# Low-energy extracorporeal shockwave therapy (ESWT) improves metaphyseal fracture healing in an osteoporotic rat model

**DOI:** 10.1371/journal.pone.0189356

**Published:** 2017-12-12

**Authors:** Gina A. Mackert, Matthias Schulte, Christoph Hirche, Dimitra Kotsougiani, Julian Vogelpohl, Bernd Hoener, Teresa Fiebig, Stefanie Kirschner, Marc A. Brockmann, Marcus Lehnhardt, Ulrich Kneser, Leila Harhaus

**Affiliations:** 1 Department of Hand-, Plastic and Reconstructive Surgery, Burn Center, Department of Plastic Surgery of the University of Heidelberg, BG Trauma Center, Ludwigshafen, Germany; 2 Department of Social- and Legal Sciences, SRH University Heidelberg, Heidelberg, Germany; 3 Department of Neuroradiology, University Medical Center Mannheim, Medical Faculty Mannheim, Mannheim, Germany; 4 Department of Neuroradiology, University Medical Center of the Johannes Gutenberg University Mainz, Mainz, Germany; 5 Department of Plastic Surgery, Burn Center, Hand Center, Sarcoma Reference Center, BG Hospital Bergmannsheil, University Hospital Bochum, Bochum, Germany; China Medical University, TAIWAN

## Abstract

**Purpose:**

As result of the current demographic changes, osteoporosis and osteoporotic fractures are becoming an increasing social and economic burden. In this experimental study, extracorporeal shock wave therapy (ESWT), was evaluated as a treatment option for the improvement of osteoporotic fracture healing.

**Methods:**

A well-established fracture model in the metaphyseal tibia in the osteoporotic rat was used. 132 animals were divided into 11 groups, with 12 animals each, consisting of one sham-operated group and 10 ovariectomized (osteoporotic) groups, of which 9 received ESWT treatment. Different energy flux intensities (0.15 mJ/mm^2^, 0.35 mJ/mm^2^, or 0.55 mJ/mm^2^) as well as different numbers of ESWT applications (once, three times, or five times throughout the 35-day healing period) were applied to the osteoporotic fractures. Fracture healing was investigated quantitatively and qualitatively using micro-CT imaging, quantitative real-time polymerase chain reaction (qRT-PCR) analysis, histomorphometric analysis and biomechanical analysis.

**Results:**

The results of this study show a qualitative and quantitative improvement in the osteoporotic fracture healing under low-energy (energy flux intensity: 0,15 mJ/mm^2^) ESWT and with fewer treatment applications per healing period.

**Conclusion:**

In conclusion, low-energy ESWT seems to exhibit a beneficial effect on the healing of osteoporotic fractures, leading to improved biomechanical properties, enhanced callus-quantity and -quality, and an increase in the expression of bone specific transcription factors. The results suggest that low-energy ESWT, as main treatment or as adjunctive treatment in addition to a surgical intervention, may prove to be an effective, simple to use, and cost-efficient option for the qualitative and quantitative improvement of osteoporotic fracture healing.

## Introduction

Due to the continuous changes in the demographic age pattern, the number of elderly people, and with it the prevalence of osteoporosis and osteoporotic fractures, is steadily increasing. In 2000, there already were an estimated 9 million fractures caused by osteoporosis worldwide and this number will continue to rise in the decades to come [1]. Due to the elevation of the prevalence of osteoporosis and its fractures, and its potentially severe consequences, the World Health Organization (WHO) has decided to list this disorder among its ten most severe diseases of current times [2]. Accompanied by a high morbidity and mortality as well as potentially resulting disability, immobility and extensive rehabilitational requirements, osteoporotic fractures have become a highly relevant clinical as well as economical issue for society and the medical health care system.

Regarding groups at risk, especially postmenopausal women are affected and have a significantly higher chance of sustaining an osteoporotic fracture during their lifetime than their male counterparts [3]. In fact, over 70% of all fractures occur in women aged 65 and older [4]. During this postmenopausal phase, a hormonal change takes place, increasing general bone-turnover and resulting in greater bone resorption than bone formation, which increases bone frailty [5]. Although there is also a certain decrease in cortical thickness, it is the deterioration of the trabecular microarchitecture, mainly located at the metaphysis, which leaves the bone in this area most prone to damage. Not only does the high incidence of osteoporotic fractures itself pose a problem, but so does the immediate and distant aftermath. Osteoporotic fracture healing is qualitatively inferior and significantly slower than normal, non-osteoporotic bone healing [6–10]. This leads to prolonged recovery periods with suboptimal bone formation and an increased risk for sustaining further fractures, possibly at the same site.

The severely diminished quality of bone in osteoporotic fractures usually requires surgical fixation with an adequate osteosynthesis technique. However, the frail microarchitecture and lowered bone mass density leads to instability and to longer healing periods, as well as related complications such as loosening or complete detachment of the hardware [11]. Therefore, an alternative or adjunct treatment option, improving this issue, is essential. The current treatment options concerning osteoporosis are limited mainly to pharmacological modalities. These primarily systemically administered medications are not only accompanied by a variety of side effects, but also by high costs and poor patient compliance [12]. Extracorporeal shock wave therapy (ESWT) could therefore present a very promising alternative to systemic medication for osteoporotic fractures, with its simple application and few to no side-effects.

The benefits of ESWT on non-osteoporotic bone have already been reported in literature [13–17]. To date, however, there has been very little published regarding the effect of ESWT on the healing of osteoporotic fractures and the existing studies have mainly examined the diaphyseal femur and tibia. However, since osteoporosis mainly affects the metaphyseal part of bone, which, contrary to the diaphyseal healing by periosteal callus formation, heals predominantly by endosteal callus formation, osteoporotic fracture healing should be investigated at the metaphyseal site [18–21]. To date, a broad range of energy flux intensities have been used, ranging from 0.0031–0.890 mJ/mm^2^, and various numbers of ESWT treatments were reported, with no consensus on an intensity or the number of treatments during the healing phase for optimal osteoporotic fracture healing [22].

The aim of this study was to investigate the effect of ESWT on the healing properties of metaphyseal fractures in a well-established osteoporotic rat model and to simultaneously investigate the optimal range of ESWT energy flux intensity and the ideal number of applications throughout the healing period able to achieve the most beneficial healing parameters regarding osteoporotic fracture healing [21].

## Materials and methods

### General procedures

The animal study protocol received approval by the local regional government. It conformed to the German animal protection laws and received permission from the District Government of Koblenz (03.04.2012, 23177-07/G 12-7-027).

132, three-month-old female Sprague-Dawley rats were obtained from Envigo (Veneray, Netherlands). 120 of these animals had undergone bilateral ovariectomy, while the remaining 12 animals were SHAM-operated, meaning they received identical procedures and incisions without removal of the ovaries. The SHAM-group served as the non-osteoporotic, positive control group. The ovariectomy-procedures were performed by Envigo (Veneray Netherlands) at an age of 2 months under isoflurane anesthesia. All rats were held under the same conditions prior to their delivery to the Ludwigshafen animal lab facilities.

Upon arrival, the 132 animals were divided into 11 study groups, each consisting of 12 animals. They were housed in standard Makrolon M-IV cages (Techniplast, Pontremoli, Italy) in non-changing groups of 3–5 animals per cage, under a 12-hour dark-light photo regimen and at 22 ± 2°C constant temperature as well as constant humidity of 50 ± 10%. The animal grouping did not change throughout the entire experiment. The animals were weighed and provided with Soy-, estrogen- and phytoestrogen-free food pellets (Ssniff, Spezialdiäten GmbH, Soest, Germany). All animals had *ad libitum* access to food and water and were allowed to move about their cages freely.

### Bilateral osteotomy and ESWT-application

**Fig 1:**
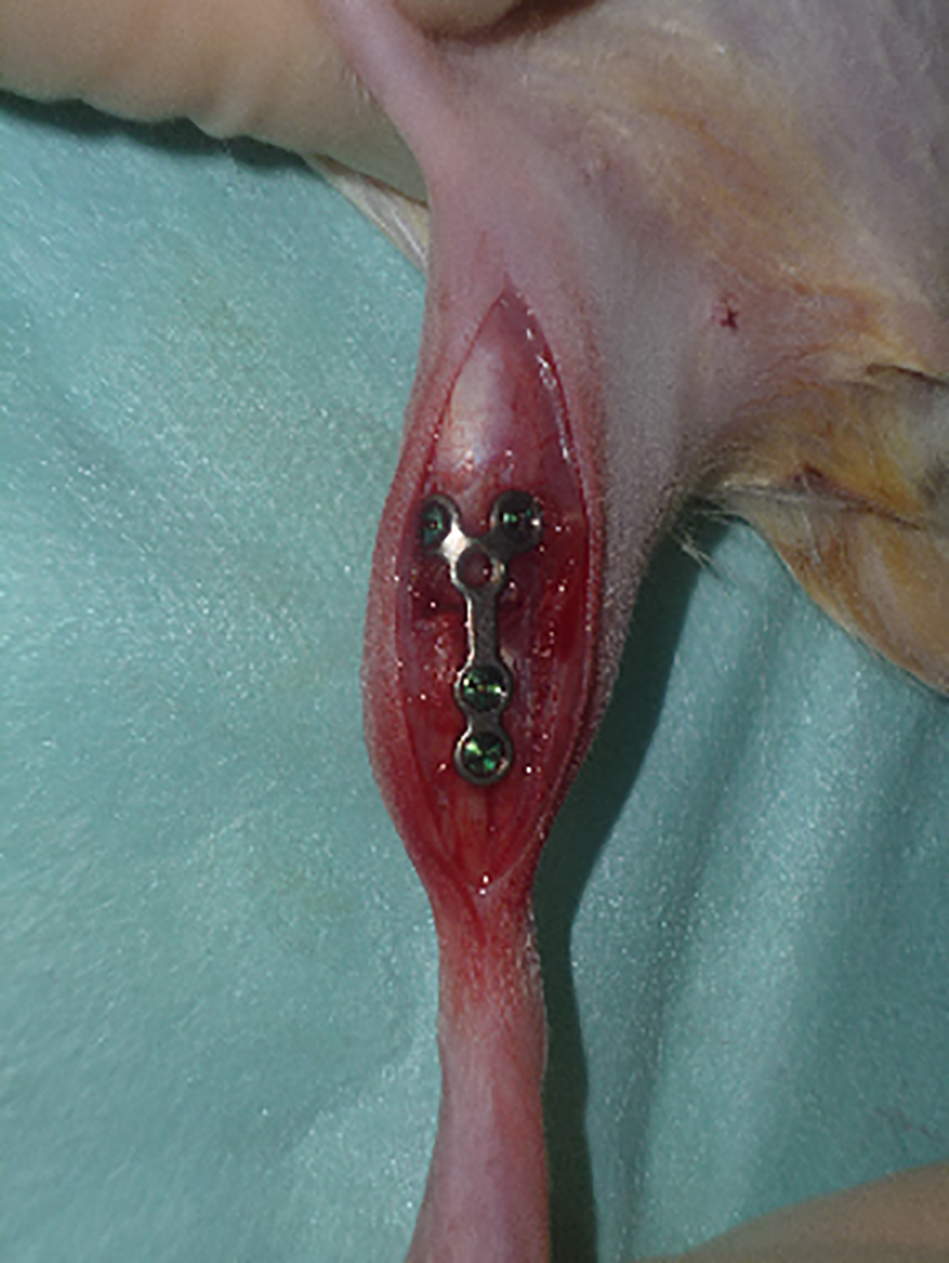
Fracture osteosynthesis of the tibia. Completed osteosynthesis of the tibia with a 5-hole mini Y-plate and 4 screws, with the plate spanning the fracture gap located in the metaphyseal area of the tibia.

Eight weeks post ovariectomy, the ovariectomized animals had developed manifest osteoporosis according to a standardized protocol [23]. The ovariectomized as well as the SHAM-ovariectomized animals then underwent a bilateral metaphyseal transverse osteotomy of the proximal *tibia* under general anesthesia with Isoflurane (2%, Isoflo, Albrecht, Aulendorf, Germany). A 1 mm osteotomy gap, 5 mm distal to the tibia plateau, was created using an oscillating, pulsed ultrasound saw (Piezosurgery^®^, Mectron Medical Technology, Cologne, Germany). The fracture fixation was conducted on the ventromedial aspect of the tibia with a 1.5 cm long and 0.6 mm profile 5-hole Y-plate (Biomet, Berlin, Germany) (Fig 1). Postoperative analgesia, consisting of Rimadyl (5mg/kg, Carprofen, Zoetis, Berlin, Germany), was weight-adjusted for each rat and administered for 2 consecutive days, with the first dose administered immediately postoperatively.

During the 35-day fracture-healing period, the animals were subjected to different regimes of ESWT according to their study-group designation. Both hindlegs of each animal received identical treatment. The ESWT applications were conducted under general anesthesia with Isoflurane. The ESWT was administered with an extracorporeal shockwave applicator (Duolith SD1, Karl Storz GmbH & Co. KG, Tuttlingen, Germany). The study design contained three different energy flux intensities (0.15 mJ/mm^2^; 0.35 mJ/mm^2^; 0.55 mJ/mm^2^) each at 2000 impulses and at 1 Hz, and three different numbers of applications of the ESWT (Table 1). The fracture healing period was set for 35 days. This time frame was chosen because callus formation at this point in time is stable, and peripheral resorption of the callus has not yet begun [21].

**Table 1:** Extracorporeal shock wave therapy application scheme.

Group (12 animals each)	Energy flux intensity (mJ/mm^2^ at 2000 impulses)	Number of extracorporeal shockwave therapy (ESWT) treatment(s)	Days of treatment(s)
SHAM	-	-	-
OVX	-	-	-
OVX (A1)	0.15 mJ/mm^2^	1	Day 1
OVX (A2)	0.15 mJ/mm^2^	3	Day 1,11,25
OVX (A3)	0.15 mJ/mm^2^	5	Day 1,8,15,22,29
OVX (B1)	0.35 mJ/mm^2^	1	Day 1
OVX (B2)	0.35 mJ/mm^2^	3	Day 1,11,25
OVX (B3)	0.35 mJ/mm^2^	5	Day 1,8,15,22,29
OVX (C1)	0.55 mJ/mm^2^	1	Day 1
OVX (C2)	0.55 mJ/mm^2^	3	Day 1,11,25
OVX (C3)	0.55 mJ/mm^2^	5	Day 1,8,15,22,29

Energy flux intensity (mJ/mm^2^) and number as well as timing of treatment(s) applied to the metaphyseal tibia fracture site according to the respective treatment group within the 35-day fracture healing period. Day 1 represents the day of the osteotomy.

### Specimen preparation

At day 35 post osteotomy, the animals were anesthetized as previously described, followed by animal sacrifice utilizing an overdose of Narcoren (Merial GmbH, Halbergmoos, Germany). Then a vertical abdominal midline incision was performed, and the uterus of each animal was harvested and weighed. Both hindlimbs of each animal were reopened, the ligaments of the knee, as well as the patellar ligament were detached from the *tibiae*. The *tibia* and *fibula* were then freed of any remaining muscle-tissue and exarticulated at the proximal and distal *tibia-fibulan* joints. The Y-plates and screws were carefully removed prior to each experiment. For the PCR samples, the callus of the randomly designated *tibiae* was collected from the fracture site and immediately frozen at -80°C. All other tibiae were frozen at -20°C until conduction of the investigations via micro-computed tomography (micro-CT) imaging, biomechanical analyses or histomorphometric analyses. From each animal study group, 6 *tibiae* were allocated to each of the four aforementioned investigations.

### Radiographic imaging and micro-computed tomography (micro-CT)

The frozen *tibiae* were thawed for three hours at room temperature. During this time the *tibiae* were subjected to radiographic imaging in an anterior-posterior and latero-medial fashion to verify the integrity of the fracture site and the alignment of the bone segments in relation to the fracture site. Immediately upon the cessation of the three hours, each *tibia* was then subjected to individual micro-computed tomography scans using an industrial micro-CT (Y.Fox; Yxlon International GmbH, Hamburg, Germany). For the evaluation of the images, the software OSIRIX (Pixmeo, SARL, Bernex, Switzerland) was used. The proximal *tibia* metaphysis above the fracture was visualized in the transverse plane, and the number of trabecular nodes present was counted. This was done as a means of confirming the presence of osteoporosis and adequate fracture alignment in the animals.

### Biomechanical analyses

The newly formed *tibial* callus was analyzed for the biomechanical parameters stiffness (S), yield load (yL), maximum load (Fmax) and failure load (fL) through a 3-point bending/breaking test using a ZWICK-testing machine (ZWICK-testing machine type Z020/TND; ZWICK-/Roell, Ulm, Germany) according to an established protocol [24, 25]. Each *tibia* was individually placed onto a friction-minimizing mobile, ball-mounted platform [24]. The stamp was aligned onto the ventral *tibia* metaphysis to apply force (Newton) precisely onto the fracture site. The feed-motion’s speed was set at 1cm/min. The trial was automatically stopped when the stamp’s linear displacement distance exceeded 0.5 cm or if the force-drop exceeded 80% of the maximum applied force. Using the software “testXpert” (Zwick GmbH & Co. KG, Ulm, Germany), the force was then graphically plotted against the travelled distance of the stamp, resulting in a curve with linear progression. Statistical evaluation of the different values for S, yL, Fmax, and fL were analyzed in an unconnected t-test. P < 0.05 was considered to be significant.

### Histomorphometric analyses

Intravital fluorochrome labeling (IFL) was used to determine the location, the amount, and the timing of callus and bone formation throughout the healing period [26]. This was done by subcutaneously injecting fluorescent dyes on pre-determined days after the osteotomy. The stains used were Xylenol orange (XO, 90 mg/kg, Sigma-Aldrich, Steinheim, Germany) applied on day 13, Calcein green (CG, green 10 mg/kg, Sigma-Aldrich, Steinheim, Germany) applied on day 18, and Alizarin red (AR, red, 30 mg/kg, Sigma-Aldrich, Steinheim, Germany) applied on days 24 and 26[26, 27].

**Fig 2:**
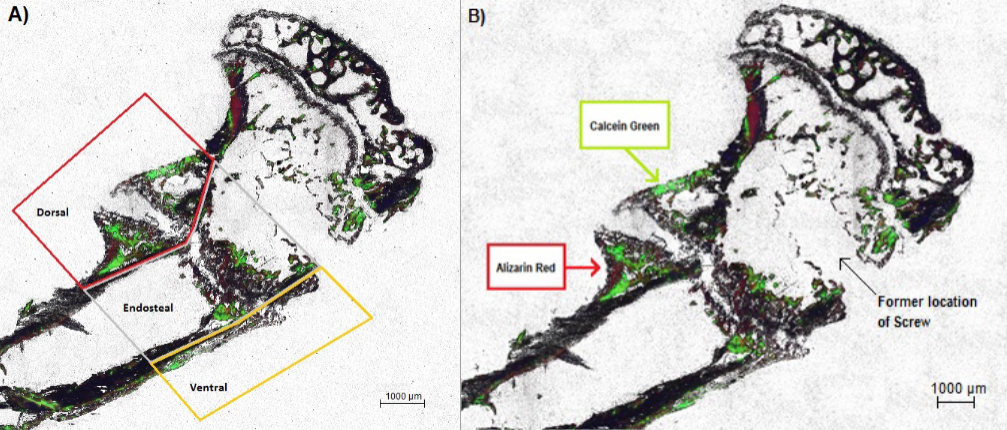
Exemplary sections of a proximal tibia from the 3 x 0.15 mJ/mm^2^ group labeled with the investigated fluorescent dyes administered previously in vivo. A) Shown is the schematic representation of the evaluated callus domains: dorsal, endosteal, and ventral. Only the callus and none of the cortical area was considered for data acquisition. B) Shown are the areas of the callus stained through the complexation of the fluorescent color with Calcium by Calcein green (green) and Alizarin red (red). The proximal, empty area represents the former location of one of the proximal osteosynthesis screws.

After micro-CT and biomechanical analysis, the *tibiae* underwent histomorphometric analysis. For this, the tibiae underwent sequential, ascending concentrations of ethanol. Subsequently they were embedded in Technovit ® 9100 methylmethacrylate (Heraeus Kulzer GmbH, Wehrheim, Germany). 10 μm longitudinal slices were cut in the *tibia’s* sagittal plane with a Microtome (Reichert Jung 2050 SuperCut Motorized Microtome, Leica Microsystems, Wetzlar, Germany) and placed on slides, then dried under pressure and 42°C heat for 72 hours. The three consecutive center sections were digitalized using a digital light microscope (Axioskop, Carl Zeiss AG, Jena, Germany) at a magnification of 100x, the analysis software Axiovision (Carl Zeiss AG, Jena, Germany) was used. The sections always included the whole proximal *tibia* and the distal *tibia* up to the distal third of the diaphysis, thereby always including the fracture site. The callus was divided into different areas of interest, namely the ventro-medial area, the endosteal area, and the dorsal area (Fig 2A). These sections were evaluated for the amount of each individual fluorochrome dye bound to the newly build callus in mm^2^ with the digital imaging software ImageJ (Fig 2B)[28, 29]. Xylenol orange represented the early period of fracture healing while Calcein green and Alizarin red represented the mid-time and late-time period of fracture healing.

### Gene expression analysis

The metaphyseal callus samples were homogenized using TriFast (VWR/PeqLab, Erlangen, Germany) and a rotor-stator-homogenizer (Heidolph, Schwabach, Germany). The total RNA was isolated using the Trizol method including DNA-digestion (RNase-free DNase Set Qiagen, Hilden, Germany). The RNA was eluted in a final volume of 30μl RNase-free H_2_O. The total RNA concentration was determined using an Eppendorf Biospectrometer (Eppendorf, Hamburg, Germany) then stored at -80°C until further processing.

Reverse transcription of the RNA samples (1 μg per sample) was conducted using the OmniScript RT Kit (Qiagen, Hilden, Germany) according to manufacturer’s instructions for first-strand synthesis using random hexamer and oligo(dT) primers. The cDNA was stored at -20°C.

Relative quantification of mRNA was performed in a two-step real-time RT-PCR procedure using the fluorescent dye SYBR Green I (LightCycler480 SYBR Green I Master, Roche, Mannheim, Germany) and a Light Cycler 480 (Roche, Mannheim, Germany). Quantitative real-time Polymerase Chain Reaction (qRT-PCR) was used to determine the following rat-genes: Osteocalcin (OC), Insulin-like Growth Factor 1 (IGF-1), Collagen 1-alpha-1 (Coll1α1), Estrogen Receptor-α (ERα), Tartrate-resistant Acid Phosphatase (TRAP), and 18-Svedberg ribosomal RNA (18S rRNA). Primer sequences are listed in Table 2. The PCR-reactions were performed with 2 μl of cDNA, 0.5 μM of sense and antisense primers and 10 μl of Lightcycler480 SYBR Green I Master reaction mix in a total volume of 20 μl. The cycling conditions were as follows: 95°C for 10 min at a ramp speed of 4,8°C/sec, 45 cycles consisting of 94°C for 15 sec at a ramp speed of 4,8°C/sec, 60°C for 10 sec at a ramp speed of 2,5°C/sec, 72°C for 10 sec at a ramp speed of 4,8°C/sec, followed by a melting point analysis: 95°C for 5 sec, 65°C for 30 sec, continuous heating to 97°C at a ramp speed of 0.1°C/sec (5 Acquisitions per second), and finally a cooling phase: 40°C for 30 sec at a ramp speed of 2°C/sec. All obtained results were corrected to 18S rRNA in each sample.

**Table 2:** Specific primer sequences used for the quantitative real-time polymerase chain reaction (qRT-PCR) amplification.

Gene	Forward Primer (5’ → 3’)	Reverse Primer (5’ → 3’)
Osteocalcin (OC)	gagggcagtaaggtggtgaa	gtccgctagctcgtcacaat
Insulin-like Growth Factor 1 (IGF-1)	ggcattgtggatgagtgttg	gtcttgggcatgtcagtgtg
Collagen1-alpha-1 (Coll1α1)	aatggtgctcctggtattgc	ggttcaccactgttgccttt
Estrogen Receptor-α (ERα)	catcgataagaaccggagga	aaggttggcagctctcatgt
Tartrate-resistant Acid Phosphatase (TRAP)	gagaacggtgtgggctatgt	gtgaagccacccagagagtc
18S ribosomal RNA (18S rRNA)	cgcggttctattttgttggt	agtcggcatcgtttatggtc

### Statistical evaluation

For statistical analysis, the mean values as well as the standard error of the mean (SEM) for each parameter were determined. Differences between the tested groups were assessed using two-way analysis of variance (SPSS Statistics Version 23; IBM Corp., New York, NY, USA) where indicated and one-way analysis of variance (SPSS Statistics Version 23; IBM Corp., New York, NY, USA), Welch-Test adjusted, as well as the Bonferroni post-hoc test. The independent variables were the number of ESWT applications during the healing period and the energy flux intensity. Adjusted p-values were considered statistically significant at p < 0.05.

## Results

### Application of ESWT

There were no adverse side effects of the ESWT application observable. The animals did not develop visible hematoma at the site of application. Immediately after the ESWT application, as soon as the anesthesia lost effect, the animals used their legs and moved about in the same pattern as they had before the application of the ESWT.

### Body weight and uterus weight

The mean body weight immediately prior to the ovariectomy and SHAM operations was 253 g ± 10 g for both groups, and did not differ significantly (p = 0.877). The mean body weight on the day of the metaphyseal tibia osteotomy was significantly different (p < 0.05) and was 275g ± 12g for the SHAM animals, and 334g *±* 24 g for the ovariectomized animals. The uterus weight at the day of sacrifice did differ significantly between the groups (p = 0.01), the SHAM-Group, with a mean uterus weight of 0.16g ± 0.1g, had a significantly higher weight than the ovariectomized animals, with a mean uterus weight of 0.07g ± 0.04g.

### Radiographic imaging and micro-computed tomography (micro-CT)

**Fig 3:**
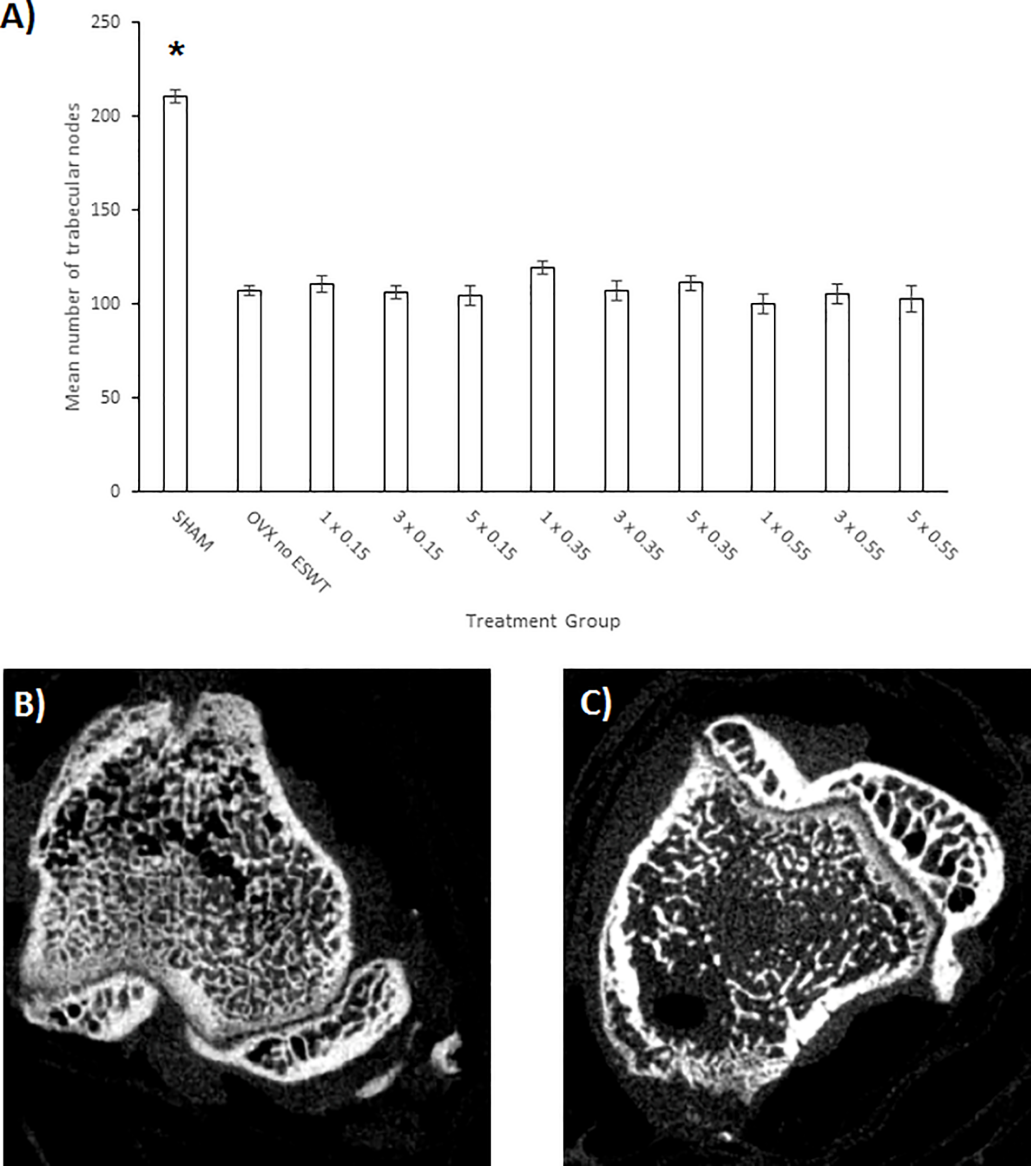
Micro-CT analyses of the number of trabecular nodes (n.Nd) in the *tibia* metaphysis above the fracture and exemplary images. A) Mean number of trabecular nodes in the proximal metaphyseal area of the tibia proximal to the fracture site according to the respective treatment group. Treatment groups are shown as number of ESWT-application(s) and energy flux intensity (mJ/mm^2^). The number of mean trabecular nodes are shown as means ± standard error of the mean (SEM) according to the respective treatment group. Adjusted p-values were considered statistically significant at p < 0.05 (Two-way analysis of variance (SPSS Statistics Version 23; IBM Corp., New York, NY, USA) and Bonferroni post hoc test). * p < 0.05 vs. all OVX groups with and without ESWT treatment. B and C) Micro-computed tomography imaging (micro-CT) of the transverse cross sections of the proximal tibia metaphysis, proximal to the fracture, and exemplary demonstration of the trabecular network with the visible difference in number of trabecular nodes (n.Nd). (B: SHAM C: OVX with ESWT treatment).

Radiographic imaging showed that there was a total of 4 *tibiae* with a fracture dislocation or pseudarthrosis in 4 different groups. These were excluded from the study. The dislocations were in the ovariectomized groups with 3 x 0.15 mJ/mm^2^, 1 x 0.55 mJ/mm^2^, 5 x 0.55 mJ/mm^2^ and the pseudarthrosis was in the group with 1x 0.35 mJ/mm^2^.The micro-CT analyses of the number of trabecular nodes (n.Nd) in the *tibia* metaphysis above the fracture showed a significant difference in the number of nodes between the ovariectomized animals with ESWT and the SHAM animals (Fig 3). There was no significant difference in the number of trabecular nodes between any of the ten ovariectomized groups. The two-way analysis of variance states that the variables seem independent of each other (p = 0,416).

### Biomechanical analysis

**Fig 4:**
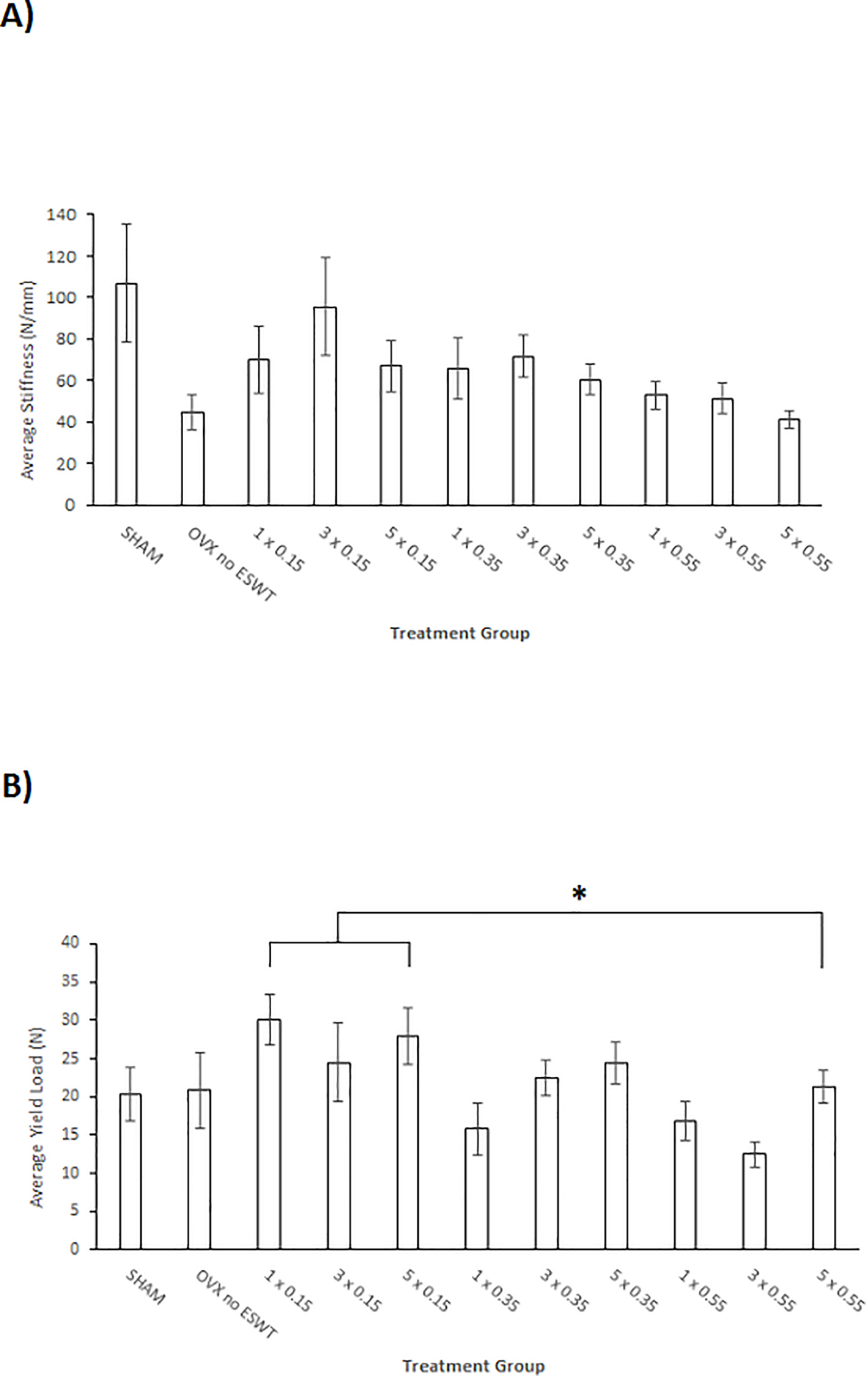
Biomechanical analysis results. Biomechanical analysis results regarding the average A) stiffness in N/mm and B) yield load in N. Both are shown against the different treatment groups (number of treatment(s) and applied energy flux intensity (mJ/mm^2^)). Values are shown as means ± standard error of the mean (SEM). Adjusted p-values were considered statistically significant at p < 0.05 (One-way analysis of variance (SPSS Statistics Version 23; IBM Corp., New York, NY, USA), Welch-Test adjusted, and Bonferroni post hoc test for stiffness; Two- way analysis of variance ((SPSS Statistics Version 23; IBM Corp., New York, NY, USA) and Bonferroni post hoc test for the other parameters). * p < 0.05 vs. 3 x 0.55 mJ/mm^2^. N: Newton, mm: milli-meter, mJ: milli-Joule.

ESWT treatment displayed a beneficial effect on the biomechanical callus properties, especially around the lower application intensities, showing an overall decrease of biomechanical properties towards the higher energy flux intensities (Fig 4). Certain ESWT treated groups even surpassed the biomechanical properties of the SHAM group. Stiffness in the ovariectomized animals was highest at 3 x 0.15 mJ/mm^2^ with 95.6 N/mm ± 23.7 N/mm SEM almost reaching the highest stiffness of 106.9 N/mm ± 28.3 N/mm SEM displayed by the SHAM group. The lowest value was found in the 5 x 0.55 mJ/mm^2^ group with 41.1 N/mm ± 4.1 N/mm SEM. The highest yield load was exhibited by the 1 x 0.15 mJ/mm^2^ group with 30.1 N ± 3.3 N SEM. There was a significant difference between the 3 x 0.55 mJ/mm^2^ group and the 1 x 0.15 mJ/mm^2^ and 5 x 0.15 mJ/mm^2^ respectively. The highest value for maximum load and failure load was exhibited by the 3 x 0.15 mJ/mm^2^ group. The lowest values for yield load, maximum load and failure load respectively were found in the 3 x 0.55 mJ/mm^2^ groups. There were no significant differences for stiffness, maximum load and failure load. In the statistical analysis it was found that there was no significance in the interaction of the two independent variables, therefore they are most likely independent of each other. A comprehensive list, including further results, is depicted in S1 Table.

### Histomorphometric analyses

**Fig 5:**
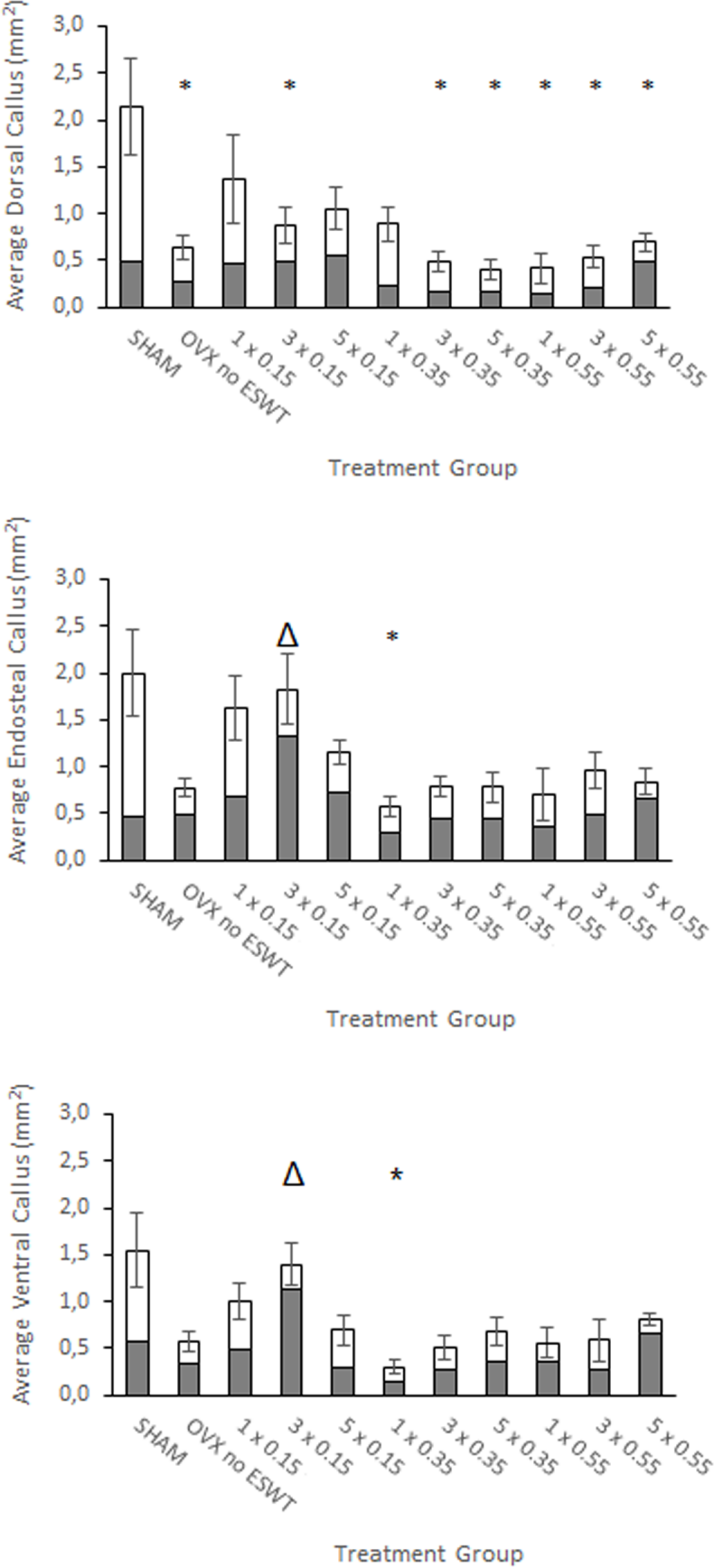
Average formed callus. Average total callus formed including simultaneous depiction of the average callus formed individually by Calcein green (white part of bar graph) and Alizarin red (grey part of bar graph). The amount of callus was measured in mm^2^ and is shown according to respective treatment group (number of treatment(s) and applied energy flux intensity (mJ/mm^2^)). Portrayed is the average total callus of the dorsal, the endosteal and the ventral aspect of the fracture callus. Values are shown as means ± standard error of the mean (SEM) of the total callus. Adjusted p-values were considered statistically significant at p < 0.05 (One-way analysis of variance (SPSS Statistics Version 23; IBM Corp., New York, NY, USA), Welch-Test adjusted, and Bonferroni post hoc test). * p < 0.05 vs. SHAM, Δ p < 0.05 vs. 1 x 0.35 mJ/mm2. mm: milli-meter, mJ: milli-Joule.

Of the three different fluorochrome colors, it was not possible to evaluate the fluorescent dye Xylenol orange as the islands formed were very small and partially overpowered by intensely green colored areas of Calcein green, and could therefore not be evaluated. Thus, we had to exclude the dye Xylenol orange from this study. The investigated fluorochrome labeled sections, Calcein green and Alizarin red, showed that most callus apposition occurred in the endosteal region, followed by the dorsal region. Least callus was formed in the ventral section of the *tibial* fracture. The dorsal callus displayed a greater area of Calcein green while in the endosteal and ventral callus, the domains stained by Alizarin red dominated slightly. In all three sections, most callus was formed in the SHAM group. The most total dorsal callus by area was found in the 1 x 0.15 mJ/mm^2^ group, while the most total endosteal and ventral callus by area was found in the 3 x 0.15 mJ/mm^2^ group. As supported by the other investigations, there was a clear increase of total callus in the lower ESWT energy flux intensities, almost entirely exceeding the ovariectomized group that did not receive ESWT treatment. There were significant differences between certain groups in all sections (Fig 5).

### Gene expression analysis

**Fig 6:**
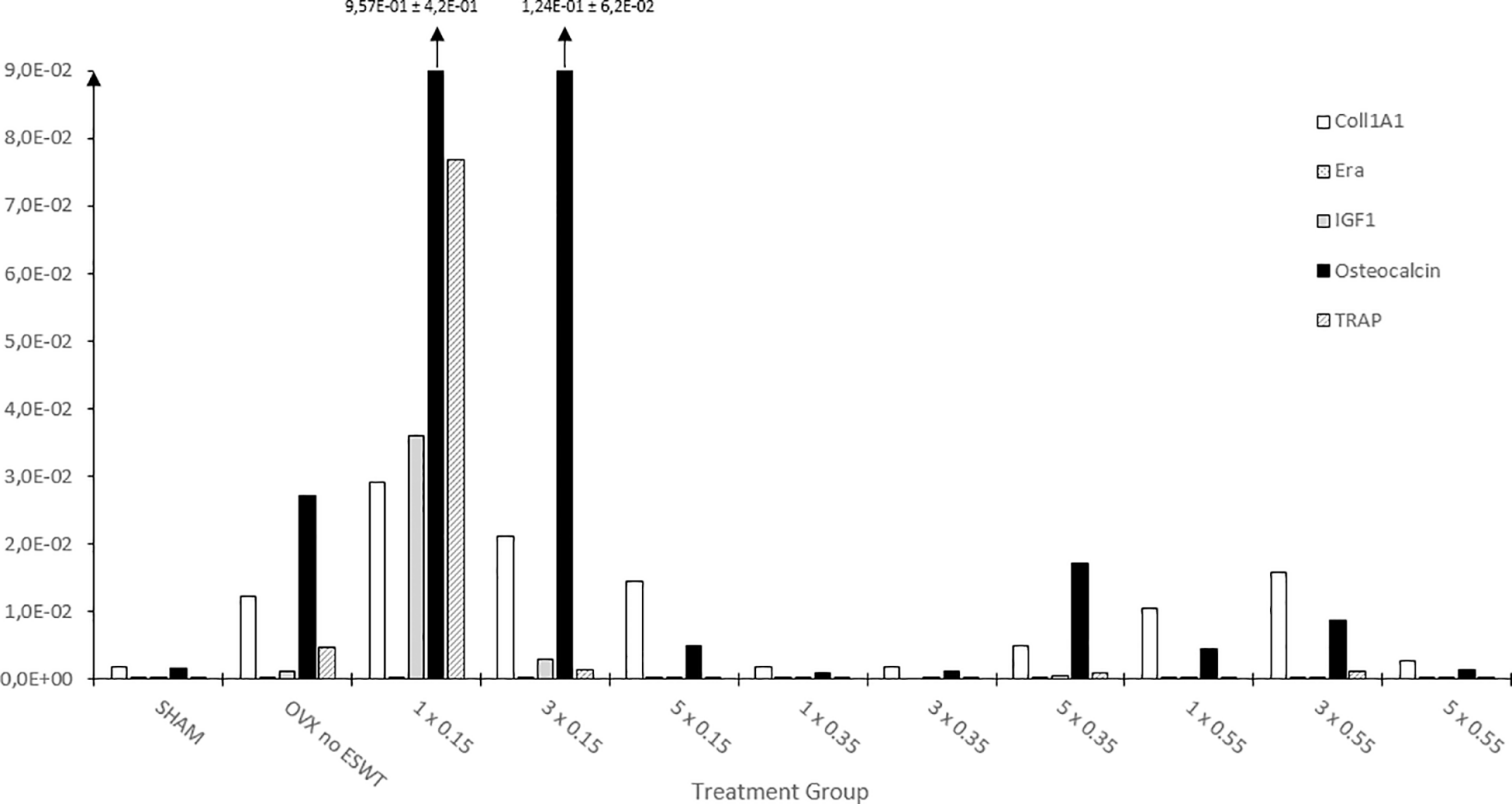
Gene expression analysis of osteoblast and osteoclast specific transcripts. Gene expression analysis through qRT-PCR of the osteoblast and osteoclast markers with regards to the respective treatment group (number of treatment(s) and energy flux intensity (mJ/mm^2^)) and shown in relative expression units. Portrayed values are shown as means ± standard error of the mean (SEM). Adjusted p-values were considered statistically significant at p < 0.05 (One-way analysis of variance (SPSS Statistics Version 23; IBM Corp., New York, NY, USA), Welch-Test adjusted, and Bonferroni post hoc test). Please see Supplement 2 for details on the numerous significances. qRT-PCR: Quantitative real-time Polymerase Chain Reaction, Coll1α1: Collagen 1-alpha-1, ERα: Estrogen Receptor-α, IGF-1: Insulin-like Growth Factor 1, OC: Osteocalcin, TRAP: Tartrate-resistant Acid Phosphatase, mJ: milli-Joule, mm: millimeter.

For all investigated transcripts (Coll1a1, ERα, IGF-1, OC, TRAP) there was a significant increase under ESWT in the lower application intensities of 0.15 mJ/mm^2^ (Fig 6). For Coll1α1, the gene expression is elevated for all application frequencies conducted with the intensity of 0.15 mJ/mm^2^. The highest value is found at 1 x 0.15 mJ/mm^2^. A second elevation can be found around the higher application intensities. For the ERα transcript, there is an increase at 1 x 0.15 mJ/mm^2^ and 3 x 0.15 mJ/mm^2^, with the highest value, measured in relative expression units, at 3 x 0.15 mJ/mm^2^ of 2,13E-02. The transcripts of IGF-1, OC and TRAP all show a significant increase at 1 x 0.15 mJ/mm^2^, with the highest value displayed by OC with 9.57E-01 relative expression units. There were significant differences among the groups for all transcripts **(S2 Table)**.

## Discussion

Within the last decades, osteoporosis has grown into a major health care issue caused by the change in population age demographics and resulting in rising health care costs and health care resource depletion. This demonstrates the importance of establishing therapy options and treatment modalities which will improve the osteoporotic fracture healing process. This study looked at the influence of focused ESWT on the metaphyseal fracture healing in an osteoporotic rat model, and found an improvement in the quantitative and qualitative healing properties of osteoporotic metaphyseal fractures of the rat tibia under low-energy ESWT.

To ensure that our model was conducted in the proper osteoporotic setting, the success of the ovariectomy was confirmed by assessing changes in body weight and uterus weight at the time of sacrifice. The body weight of the OVXs animals was significantly higher compared to the SHAM animals, which is indicative of a successful ovariectomy [30]. Additionally, the uterus weight of the ovariectomized animals was significantly lower than that of the SHAM animals, which can be attributed to the lack of hormonal uterus-tissue stimulation, again in accordance with a successful ovariectomy. Further, micro-CT evaluation of the number of trabecular nodes (n.Nd) showed the typical bone loss in the trabecular network (trabecular nodes) with a significant loss of trabecular nodes in the ovariectomized animals compared to the SHAM animals (Fig 3). Therefore, the integrity of the osteoporotic rat model was verified.

The mechanism of action with which ESWT influences bone is not yet fully understood and remains subject of discussion. The positive influence of ESWT on fractures was initially attributed to the induction of microtraumata, microlesions and the formation of haematoma, which was thought to induce neo-vascularization and activate osteoblasts and fibroblasts [13, 31, 32]. More recent literature showed that neovascularization is caused through increased expression of angiogenetic growth factors such as endothelial nitric oxide synthase (eNOS), vascular endothelial growth factor (VEGF) and proliferating cell nuclear antigen (PCNA) in tendons, bone and the tendon-bone transitions. Furthermore, there is a direct activation of fibroblasts and subsequent transformation into osteoblasts [33, 34]. The expression of different mediators as the mechanism of action of extracorporeal shock waves is hypothesized to originate in the concept of mechanotransduction[35, 36]. It is assumed to be responsible for the activation of different genes and intracellular signal transduction pathways [37]. For example, Xu *et al*. have found that the expression of a transmembrane protein, the integrin α5ß1, found on the surface of osteoblasts, is elevated in response to extracorporeal shock wave treatment [37]. Our study did not intend to investigate the mechanism of action, but rather the influence of ESWT on osteoporotic fracture healing.

The results of this study found that focused low-energy ESWT has a beneficial effect on the quantitative and qualitative healing properties and characteristics of osteoporotic fractures when compared to osteoporotic fractures without ESWT treatment. The biomechanical properties showed an improvement under low-energy (energy flux intensity 0.15 mJ/mm^2^) ESWT. Van der Jagt *et al*., using an energy flux intensity of 0.16mJ/mm^2^, also found that ESWT treatment led to significantly improved biomechanical properties of osteoporotic bone (without fracture) in an osteoporotic rat model. They found an increase in the stiffness of 10% and an increase of the maximum load of 14% when compared to non-osteoporotic bone [38]. Chen *et al*. had similar results in their osteoporotic *tibia* metaphyseal fracture rat model using an energy flux intensity of 0.25 mJ/mm^2^. They found a significant increase in the maximum load (p < 0.05) and a 25.5% increase in the failure load under high energy shock wave treatment [39].

The investigation of the trabecular nodes found in the micro-CT analysis showed that there was a significantly lower number of trabecular nodes in the ovariectomized groups than in the in the SHAM group (p < 0.05). There was no significant difference in the number of trabecular nodes between the ovariectomized groups themselves (Fig 3). Further parameters of the micro-CT analysis were not investigated, however it would be interesting to see in future experiments, under a comprehensive micro-CT analysis, if there is a significant difference in the trabecular network between ovariectomized animals with and without ESWT treatment. If there is no significant difference in the trabecular network between the osteoporotic groups with and without ESWT, it should be further investigated if ESWT within the examined time period solely exert an influence on the callus and not on the trabecular network. This could pose the question whether ESWT could be used for treating osteoporotic fractures, but may not exhibit a beneficial effect when used for treating osteoporosis in osteoporotic bone. In literature, Chen *et al*., in a *tibial* fracture model in osteoporotic rats, found a 20.9% higher average trabecular thickness in the ovariectomized group treated with ESWT (0.26 mJ/mm^2^) than in the ovariectomized control group not treated with ESWT [39]. They also found a 28.4% higher number of trabeculae in the treatment group compared to their control group. Van der Jagt *et al*. found, in an osteoporotic rat model without a fracture, that ESWT (0.3 mJ/mm^2^) led to a higher trabecular thickness and a lower connectivity density of the trabeculae in the group treated with ESWT than in the ovariectomized control group without ESWT treatment [38]. The differences were not significant however. The heterogeneity of results indicates that there is a need for further investigation regarding ESWT and the effect on the trabecular architecture.

Regarding callus quantity, this study found an increase in callus formation under low-energy ESWT, especially in the endosteal and dorsal areas. By the appositional pattern of Alizarin Red, it has been shown that there was more callus formed around days 24 and 26, than by apposition of Calcein Green around day 18. Chen *et al*., using 0.26 mJ/mm^2^, also found greater callus formation in the treated group than in the control group. In fact, the callus volume was 20.1% (p < 0.05) higher in the fracture group treated with high energy shock waves (HESW) than the ovariectomized group without treatment [39]. They did not, however, investigate the three sections (ventral, endosteal, dorsal) separately. Their study used intramedullary pins for fracture osteosynthesis, compared to the Y-plate system utilized in this study. This prevents full endosteal callus formation, and may have an impact on the mechanical environment influencing callus formation. Van der Jagt *et al*. also found that unfocused extracorporeal shock waves (UESW) have a beneficial effect on osteoporotic bone [40]. In their experiment, they used an energy flux intensity of 0.16 mJ/mm^2^ on the metaphyseal *tibia* in an osteoporotic rat model and found that 3 weeks after ESWT treatment, the trabecular volume was 110% of the baseline value, while the control group only displayed 101% of the initial baseline value (p = 0.001). However, they did not find the UESW therapy to have any effect on the fibular osteotomy regarding bone morphometric parameters. The said study, however, was done with unfocused ESWT and a *fibular* fracture model. Further investigations are necessary to analyze the influence of different study design parameters on the outcome.

This study also investigated osteoblast and osteoclast specific transcription factors and found that there was an increase in their gene-expression under low-energy ESWT therapy. There were increases in all tested osteoblast transcription factors, OC, IGF-1, Coll1α1, ERα, as well as in the tested osteoclast transcription factor TRAP, showing that both cell types were activated by the application of ESWT, supporting the idea that ESWT may have a fundamental influence leading to the activation of complex pathways within the cells. In current literature, the influence on gene expression of ESWT applied to osteoporotic fractures has to date not been sufficiently investigated. In a non-osteoporotic bone defect in a rat model, Chen *et al*. found, under ESWT with an energy flux density of 0.16 mJ/mm^2^, mesenchymal stem cells were recruited and the expression of TGF-ß1 and VEGF-A in the regenerating bone tissue was significantly increased [41]. Ma *et al*., in an experiment looking at necrotic femoral heads in a rabbit model, found an increase in gene expression of BMP-2 under ESWT application [42]. Wang *et al*. also found an increase in BMP-2, VEGF, PCNA and eNOS in a rabbit study treating non-osteoporotic long bone fractures with ESWT [16]. Further Wang *et al*. found, that the serum levels of BMP-2, VEGF, PCNA and eNOS in bony non-unions treated with ESWT in a clinical study were significantly elevated [43]. An *in vitro* study done by Hofmann *et al*. looking at the influence of shock waves on human osteoblasts, did not find any change in the expression of TGF-ß1, Osteocalcin, Osteopontin, or Osteonectin. They did however find an increase of PTHrP and PGER3, factors important for osteoblast differentiation [44]. Most of the literature published supports our finding that low-energy ESWT stimulate an increase in gene expression and thus transcription factors important to bone and fracture healing. Since the evidence of the influence of ESWT on osteoporotic bone and fracture healing to date is underrepresented, again, further investigations are necessary.

So far varying strengths of energy flux intensities have been used in past studies, ranging from 0.003–0.890 mJ/mm^2^ [22]. Studies have shown that biological responses are initiated at around 0.15 mJ/mm^2^ and 500 pulses [41, 45, 46]. In this study, the lower energy flux intensities (0.15 mJ/mm^2^) had a more beneficial effect on the biomechanical properties, the amount of formed callus and the gene expression than did the higher energy flux intensities. The influence of the number of applications (once, three-times, five-times) did not seem to have as much of a notable influence on the osteoporotic fracture healing, suggesting that the important parameter influencing the quality and quantity of osteoporotic fracture healing is the energy flux intensity with which the fracture is treated. Looking at the results, the optimum beneficial range for osteoporotic fracture healing seems to lie in the combination of lower energy flux intensities, such as 0.15 mJ/mm^2^ and lower numbers of one to three applications during the fracture healing period. Energy levels of 0.54–0.9 mJ/mm^2^ have been shown to cause trabecular disruption and fracturing of the cortex, supporting our finding of the effectiveness of the lower energy intensities [32, 47]. Higher energy flux intensities, as well as higher numbers of applications thus seem to be rather detrimental to the cause, and was in concordance with our results. This may be due to the potential physical and mechanical damage done to the trabecular network and the newly formed callus and bone, accompanied by the subsequent possible interruption of the healing process.

There are certain limitations to our study. A limitation in this study is the fact that we were not able to evaluate the areas of the callus stained by Xylenol orange. In prior, preliminary studies that have been conducted, no problems evaluating this fluorescent dye were encountered. A possibility explaining the lack of early xylenol orange labeled bone could be the result of bone turnover by the time the specimens were collected and examined after 35 days of fracture healing. One possible cause may be the elevation of TRAP levels (especially as seen in the 1 x 0.15 mJ/mm^2^ group), which would have to be investigated in further studies. Since it was not possible to evaluate the callus stained by Xylenol orange in this study, we are not able to objectively make any assumptions regarding the very early phase of callus formation. A further limitation of this study is that energy intensities lower than 0.15 mJ/mm^2^ were not examined. This also needs to be investigated in further studies to see if there is a beneficial effect on the callus formation and fracture healing below our lowest intensity of 0.15 mJ/mm^2^.

In summary, this study suggests that low-energy ESWT has a beneficial effect on the metaphyseal, *tibial* osteoporotic fracture healing in an osteoporotic rat model. This treatment modality led to an improvement of biomechanical properties, histomorphometrical parameters, also osteoblast and osteoclast activity. Further, the study suggests that the beneficial spectrum of energy flux intensities lies in the lower energy flux intensities, for example, as found in this study, around 0.15 mJ/mm^2^. These results support the experimental basis for ESWT use. Translating these beneficial ESWT tendencies into clinical practice through further studies, this treatment potentially could, as either a single therapy modality or as an adjunctive treatment option, improve osteoporotic fracture healing, with the group of focus represented by especially the elderly population, maybe leading to faster and qualitatively superior callus properties. Furthermore, ESWT is simple to apply, cost efficient and has few to no side effects. Additional studies are needed, but it could hold potential for providing a new treatment modality, possibly improving the qualitative and quantitative outcome of the osteoporotic fracture healing process, potentially leading decreased patient morbidity and mortality as well as decreased health care costs.

## Supporting information

S1 Table Extended biomechanical analysis results. Results of the four different biomechanical investigations stiffness (N/mm), yield load (N), maximum load (N), and failure load (N) according to the respective number of treatment(s) and energy flux intensity (mJ/mm^2^). Values are shown as means ± standard error of the mean (SEM). Adjusted p-values were considered statistically significant at p < 0.05 (One-way analysis of variance (SPSS Statistics Version 23; IBM Corp., New York, NY, USA), Welch-Test adjusted, and Bonferroni post hoc test for stiffness; Two- way analysis of variance ((SPSS Statistics Version 23; IBM Corp., New York, NY, USA) and Bonferroni post hoc test for the other parameters) * p < 0.05 vs. 3 x 0.55 mJ/mm^2^. N: Newton, mJ: milli-Joule, mm: millimeter.
(TIF)

S2 Table Extended gene expression analysis results of osteoblast and osteoclast specific transcripts. Gene expression of the investigated osteoblast and osteoclast specific transcription markers through qRT-PCR. The portrayed values are in relative expression units and shown as means ± Standard Error of the Mean (SEM) respective to their treatment group (number of treatment(s) and energy flux intensity (mJ/mm^2^)). Adjusted p-values were considered statistically significant at p < 0.05 (One-way analysis of variance (SPSS Statistics Version 23; IBM Corp., New York, NY, USA), Welch-Test adjusted, and Bonferroni post hoc test). † p < 0.05 vs. SHAM, * p < 0.05 vs. 1 x 0.15 mJ/mm^2^, Δ p < 0.05 vs. 3 x 0.15 mJ/mm^2^. qRT-PCR: Quantitative real-time Polymerase Chain Reaction, Coll1α1: Collagen 1-alpha-1, ERα: Estrogen Receptor-α, IGF-1: Insulin-like Growth Factor 1, OC: Osteocalcin, TRAP: Tartrate-resistant Acid Phosphatase, mJ: milli-Joule, mm: millimeter.
(TIF)
