# Supplementary figures and images for: Low-energy extracorporeal shockwave therapy (ESWT) improves metaphyseal fracture healing in an osteoporotic rat model
Source: PLoS One. 2017 Dec 12;12(12):e0189356. doi: 10.1371/journal.pone.0189356 (PMC5726728; doi:10.1371/journal.pone.0189356)

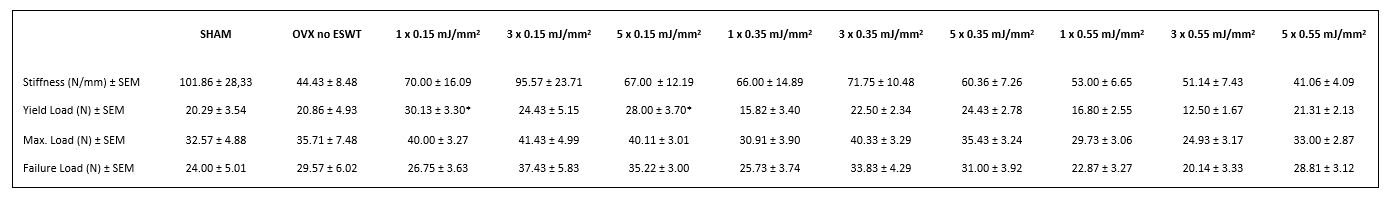

Supplement: S1 Table — Results of the four different biomechanical investigations stiffness (N/mm), yield load (N), maximum load (N), and failure load (N) according to the respective number of treatment(s) and energy flux intensity (mJ/mm2). Values are shown as means ± standard error of the mean (SEM). Adjusted p-values were considered statistically significant at p < 0.05 (One-way analysis of variance (SPSS Statistics Version 23; IBM Corp., New York, NY, USA), Welch-Test adjusted, and Bonferroni post hoc test for stiffness; Two- way analysis of variance ((SPSS Statistics Version 23; IBM Corp., New York, NY, USA) and Bonferroni post hoc test for the other parameters) * p < 0.05 vs. 3 x 0.55 mJ/mm2. N: Newton, mJ: milli-Joule, mm: millimeter. (TIF) [file pone.0189356.s001.tif]

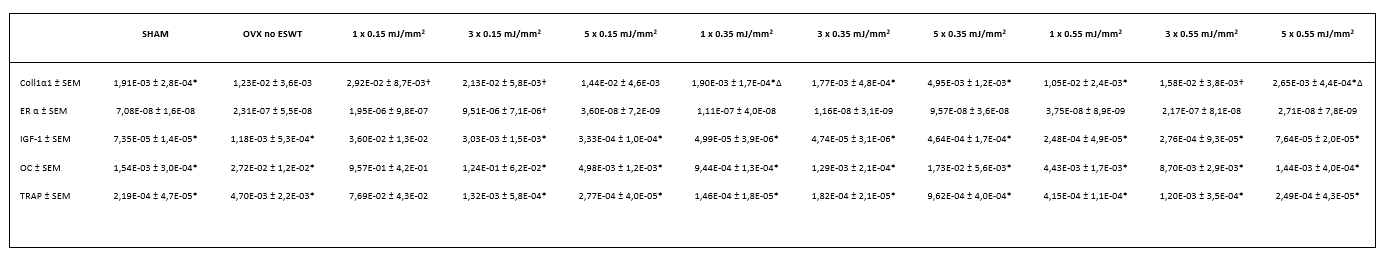

Supplement: S2 Table — Gene expression of the investigated osteoblast and osteoclast specific transcription markers through qRT-PCR. The portrayed values are in relative expression units and shown as means ± Standard Error of the Mean (SEM) respective to their treatment group (number of treatment(s) and energy flux intensity (mJ/mm2)). Adjusted p-values were considered statistically significant at p < 0.05 (One-way analysis of variance (SPSS Statistics Version 23; IBM Corp., New York, NY, USA), Welch-Test adjusted, and Bonferroni post hoc test). † p < 0.05 vs. SHAM, * p < 0.05 vs. 1 x 0.15 mJ/mm2, Δ p < 0.05 vs. 3 x 0.15 mJ/mm2. qRT-PCR: Quantitative real-time Polymerase Chain Reaction, Coll1α1: Collagen 1-alpha-1, ERα: Estrogen Receptor-α, IGF-1: Insulin-like Growth Factor 1, OC: Osteocalcin, TRAP: Tartrate-resistant Acid Phosphatase, mJ: milli-Joule, mm: millimeter. (TIF) [file pone.0189356.s002.tif]
